# Nomogram Predicting Cancer-Specific Death in Parotid Carcinoma: a Competing Risk Analysis

**DOI:** 10.3389/fonc.2021.698870

**Published:** 2021-10-13

**Authors:** Xiancai Li, Mingbin Hu, Weiguo Gu, Dewu Liu, Jinhong Mei, Shaoqing Chen

**Affiliations:** ^1^ Department of Oncology, The First Affiliated Hospital of Nanchang University, Nanchang, China; ^2^ Department of Burn, The First Affiliated Hospital of Nanchang University, Nanchang, China; ^3^ Department of Pathology, The First Affiliated Hospital of Nanchang University, Nanchang, China

**Keywords:** SEER, parotid cancer, cancer-specific death, competing risk, nomogram

## Abstract

**Purpose:**

Multiple factors have been shown to be tied to the prognosis of individuals with parotid cancer (PC); however, there are limited numbers of reliable as well as straightforward tools available for clinical estimation of individualized mortality. Here, a competing risk nomogram was established to assess the risk of cancer-specific deaths (CSD) in individuals with PC.

**Methods:**

Data of PC patients analyzed in this work were retrieved from the Surveillance, Epidemiology, and End Results (SEER) data repository and the First Affiliated Hospital of Nanchang University (China). Univariate Lasso regression coupled with multivariate Cox assessments were adopted to explore the predictive factors influencing CSD. The cumulative incidence function (CIF) coupled with the Fine-Gray proportional hazards model was employed to determine the risk indicators tied to CSD as per the univariate, as well as multivariate analyses conducted in the R software. Finally, we created and validated a nomogram to forecast the 3- and 5-year CSD likelihood.

**Results:**

Overall, 1,467 PC patients were identified from the SEER data repository, with the 3- and 5-year CSD CIF after diagnosis being 21.4% and 24.1%, respectively. The univariate along with the Lasso regression data revealed that nine independent risk factors were tied to CSD in the test dataset (*n* = 1,035) retrieved from the SEER data repository. Additionally, multivariate data of Fine-Gray proportional subdistribution hazards model illustrated that N stage, Age, T stage, Histologic, M stage, grade, surgery, and radiation were independent risk factors influencing CSD in an individual with PC in the test dataset (*p* < 0.05). Based on optimization performed using the Bayesian information criterion (BIC), six variables were incorporated in the prognostic nomogram. In the internal SEER data repository verification dataset (*n* = 432) and the external medical center verification dataset (*n* = 473), our nomogram was well calibrated and exhibited considerable estimation efficiency.

**Conclusion:**

The competing risk nomogram presented here can be used for assessing cancer-specific mortality in PC patients.

## Introduction

Parotid cancers are responsible for about 70% of malignant tumors in the salivary gland, characterized by pathological/histological differences (1, 2). The present crude incidence of primary cancers of the salivary is 0.9 per 100,000, of which approximately 80% of these cases arise in the parotid salivary gland (3). The prognosis of individuals with PC differs significantly, with some clinical features considerably influencing the disease-free survival (DSF) along with (OS) overall survival. Its mortality rate has remained the same over the past decade, with a 5-year OS of approximately 60% dependent on the histological type, as well as the anatomical site, and specifically the treatment option (4, 5). Presently, the AJCC staging criteria are the main approach to estimating prognosis in individuals with parotid cancer. Nonetheless, remarkable differences in the clinical outcomes among individuals with parotid cancer at the same stage receiving similar treatments have been reported (6). This demonstrates that the AJCC staging method is far from being a perfect system for making a prognosis, as well as treatment decisions. Such a method is only ideal for estimating distant metastasis (M stage), tumor size along with extension (T stage), and lymph node (LN) involvement without taking into account other factors, e.g., histological types, demographical factors, and treatments. Recently, numerous researches have documented the prognosis of common head and neck cancer, such as laryngeal carcinoma (7) and nasopharyngeal carcinoma (8), but few have addressed PC. The survival of individuals with PC has been investigated by other research groups; nonetheless, most investigations are from single institutions lacking the assessment of CSD risk factors. Hence, it is pivotal to conduct more research on PC prognosis.

Surveillance, Epidemiology, and End Results (SEER), a data repository based on populations, represents an estimated 28% of the US population. Therefore, the datasets retrieved from the SEER data repository provide adequate cases of creating prognostic models, particularly for rare cancers (9). The data of PC cases utilized in this research were retrieved from the SEER data repository, which can guarantee the authenticity and sufficiency of the data. Overall, cancer patients frequently experience more than two events, but only one event occurs (10). The events excluding that of interest are termed as competing risks. In traditional survival assessment, censoring of competing risks is done and can be enhanced through competing risk assessment.

A nomogram visualizes the linear prognosis of a disease (11). Each characteristic value on the nomogram plot signifies a score, with the total score mapping the survival estimate. In many studies, survival outcomes are determined using the Kaplan-Meier approach coupled with the Cox proportional hazard, although the population-based approaches are also applied (12, 13). Nonetheless, a significance of the studies analyzed the OS along with the cancer-specific survival assessment, while neglecting the role played by other competing causes of death in the prognosis of nonmetastatic PC. Prolonged survival is dependent on the competing risks of death to a remarkable degree. The competing risk should be considered when forecasting survival outcomes.

In this work, we aimed to construct a competing risk nomogram using data retrieved from the SEER data repository to assist in predicting death linked to PC. The nomogram will help clinicians in making patient-specific decisions in treating PC as well as precise predictions of disease outcomes.

## Material and Methods

### SEER Database Patients

We retrospectively analyzed data from the SEER data repository spanning from 1992 to 2017. The SEER data repository (https://seer.cancer.gov/) is publicly accessible.

A selection of SEER 13 Regs Custom Data (with additional treatment fields) uploaded in November 2019 (1992–2017 varying) was done. All subjects with primary PC diagnosis (site recode NM7/CS v0204+ Schema of “parotid gland” along with the ICD-O-3 behavior recode of “malignant”) were enrolled in the analysis. Exclusion criteria consisted of PC individuals who were less than 5 years old, those with a survival time of ≤1 month, and patients lacking complete data or a pathological diagnosis.

### Our Medical Center Patients

We collected data from 473 individuals with PC who were admitted to the First Affiliated Hospital of Nanchang University (China) spanning from 2006 to 2017. The subjects confirmed by pathology had no history of other malignant tumors. The Ethics Committee of the First Affiliated Hospital of Nanchang University approved this retrospective cohort study. The principles of the Helsinki Declaration were followed with regards to data confidentiality.

### Variable Selection

Factors including age, T stage, AJCC stage, N stage, race, M stage, sex, histological type, surgery, radiation, grade, follow-up time, and survival outcomes were retrieved from the SEER data repository. We adopted the X-tile software to determine the optimal threshold values. The age of the subjects at diagnosis was classified into two classes, i.e., ≥70 and <70 years. The AJCC stage was employed as the staging approach. The ICD-O-3 codes were adopted to categorize the PC histological type into two classes, i.e., mucoepidermoid carcinoma (MEC) and none MEC (squamous cell carcinoma, myoepithelial carcinoma, polymorphous adenocarcinoma NOS, adenoid cystic carcinoma, and acinic cell carcinoma, among others) as per the WHO categorization approach. Cancer-distinct survival constituted the primary end-point and included the time beginning from cancer diagnosis to death emerging from PC or a censored risk. Deaths linked to accidents or diseases excluding PC constituted the competition risks.

### Statistical Analyses

We conducted all the analysis using R (V.4.0.4: survivial, crrstep, cmprsk, pec, rms, riskRegression, mstate, and foreign packages) to perform the statistical analyses. Two-sided and *p* < 0.05 defined statistical significance. Firstly, we computed the CIF for 3 to 5 years. We further carried out subgroup analysis between diverse subgroups, and matching CIF curves were created for these variables. Gray’s test was implemented to determine the drastic differences in values of CIF among subgroups. Secondly, patients from the SEER data repository were split at random into a test data set along with the verification data set, with a 7:3 ratio. Patients recruited from our hospital served as the external verification dataset. The test dataset was employed to create the prediction nomogram for estimating of CSD, whereas the verification datasets were employed to validate the efficiency of our nomogram. Univariate coupled with the Lasso Cox regression model assessments were implemented to explore the independent predictors of CSDs in the test dataset. All different variables were further identified by AIC and BIC models. The Fine-Gray proportional hazards model was adopted to develop the competing risk nomogram.

The performance of our nomogram was first explored in the test cohort and subsequently in the verification cohorts with respect to the C-index, AUC, and the calibration curve. The estimation capacity of our nomogram was quantified with the C-index and ranged from 0.5 to 1.0, representing a random probability from indicating no discrimination to indicating optimal discrimination (14). The AUC reflects the overall estimation value for all the thresholds (15), with a perfect prediction value exhibiting an AUC of 1.0. We adopted decision curve analysis (DCA) to determine the clinical net benefit of different probability thresholds for a possible clinical consequence (16) and explored the nomogram efficiency in contrast with the AJCC staging approach visually.

## Results

### Baseline Features of Participants

**Figure 1:**
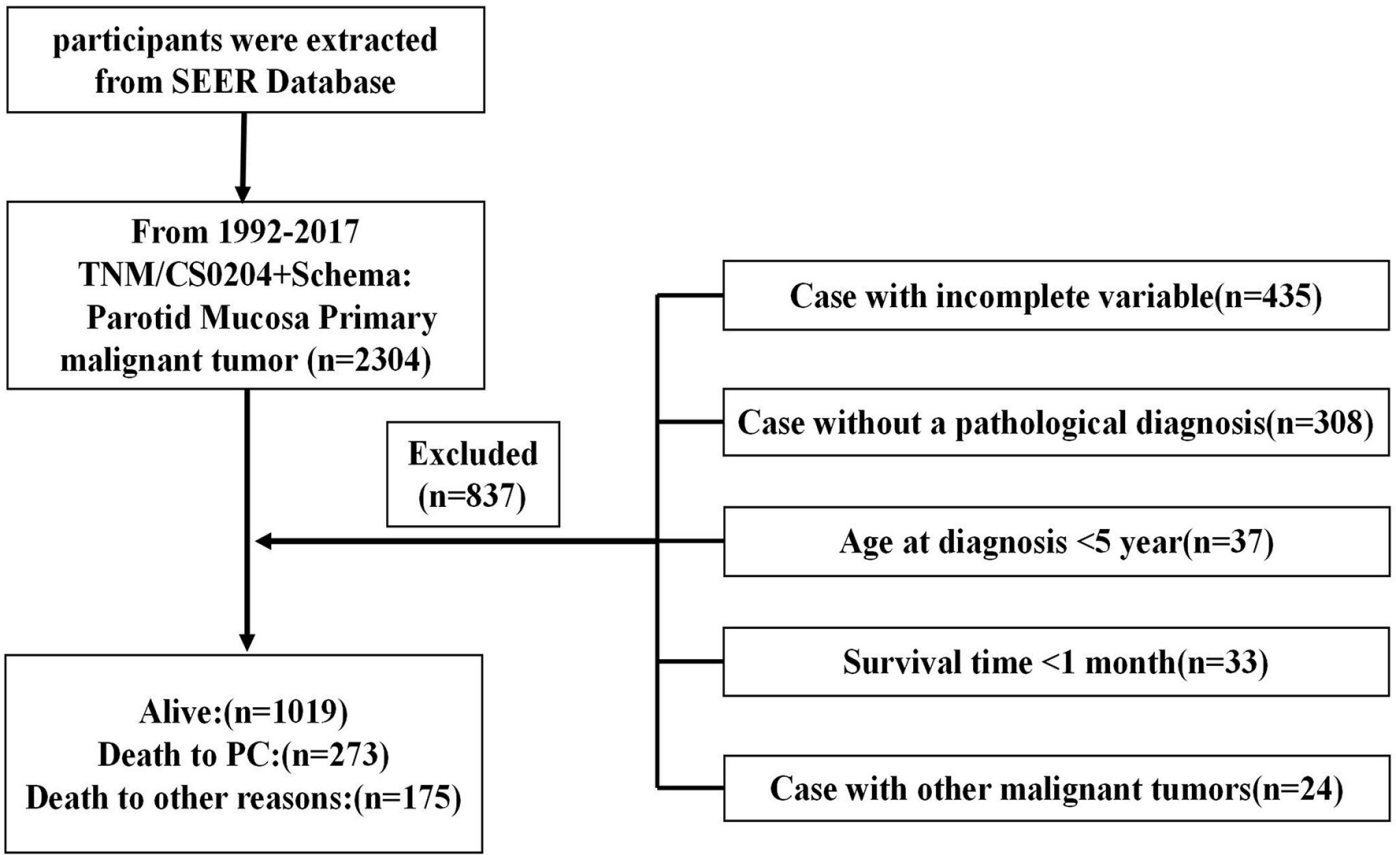
Flow diagram illustrating recruitment of patients. SEER, Surveillance, Epidemiology, and End Results; PC, parotid cancer.

As illustrated in **Figure 1**, we initially retrieved 2,304 patient cases from the SEER data repository. Strict screening was carried out, yielding 1,467 patient cases with PC who were recruited in the study. The subjects’ median age was 50.7 years (5–85) at diagnosis with males accounting for 43.6%. Most participants were white (*n* = 1,142, 77.8%). Of the 1,467 PC cases, 621 (42.3%) were MEC, consisting of 599 (40.8%) incidences of moderate differentiation. Besides, stage I constituted the most frequent tumor stage (*n* = 454, 30.9%), followed by stages IV (*n* = 420, 28.6%), II (*n* = 334, 22.8%), and III (*n* = 259, 17.7%). Most PC subjects were classified as T1 (35.0%), followed by T2 (28.9%), T3 (19.9%), and T4 (16.2%). More than half of the PC subjects lacked lymph node (LN) metastases (N0, 72.1%), and most patients did not exhibit distant metastases (M0, 96.4%). A significant number of the PC individuals were treated using surgical therapy (*n* = 1,375, 93.7%) and 35.5% of the patients received radiotherapy. The detailed demographic, as well as clinical characteristics of the recruited participants, are given in **Table 1**.

**Table 1:** Basic characteristics of parotid cancer patients in the training, internal validation, and external validation cohorts.

Characteristics	SEER database		Our medical center
	Training cohort (*n* = 1,035)	Internal validation cohort (*n* = 432)	External validation cohort (*n* = 473)
	*n* (%)	*n* (%)	*n* (%)
Age (years)			
<70	689 (66.6)	291 (67.4)	416 (87.9)
≥70	346 (33.4)	141 (32.6)	57 (12.1)
Race			
White	795 (76.8)	347 (80.3)	
Black	93 (9.0)	37 (8.6)	
Others	147 (14.2)	48 (11.1)	473 (100.0)
Sex			
Male	446 (43.1)	193 (44.7)	261 (55.2)
Female	589 (56.9)	239 (55.3)	212 (44.8)
AJCC stage			
I	331 (32.0)	123 (28.5)	123 (26)
II	228 (22.0)	106 (24.5)	184 (38.9)
III	176 (17.0)	83 (19.2)	121 (25.6)
IV	300 (29.0)	120 (27.8)	45 (9.5)
T stage			
T1	365 (35.3)	149 (34.5)	119 (25.2)
T2	291 (28.1)	134 (31.0)	201 (42.5)
T3	206 (19.9)	86 (19.9)	126 (26.6)
T4	173 (16.7)	63 (14.6)	27 (5.7)
N stage			
N0	747 (72.2)	311 (72.0)	375 (79.3)
N1	110 (10.6)	49 (11.3)	61 (12.9)
N2	178 (17.2)	72 (16.7)	37 (7.8)
M stage			
M0	997 (96.3)	417 (96.5)	462 (97.7)
M1	38 (3.7)	15 (3.5)	11 (2.3)
Surgery			
No	64 (6.2)	28 (6.5)	14 (3.0)
Yes	971 (93.8)	404 (93.5)	459 (97.0)
Grade			
Well	241 (23.3)	94 (21.8)	207 (43.8)
Moderate	422 (40.8)	177 (41.0)	89 (18.8)
Poorly/undifferentiated	372 (35.9)	161 (37.3)	177 (37.4)
Radiation			
No	668 (64.5)	278 (64.4)	263 (55.6)
Yes	367 (35.5)	154 (35.6)	210 (44.4)
Histologic type			
MEC	445 (43)	176 (40.7)	185 (39.1)
No-MEC	590 (57)	256 (59.3)	288 (60.9)

### CIF Survival Analysis

**Figure 2:**
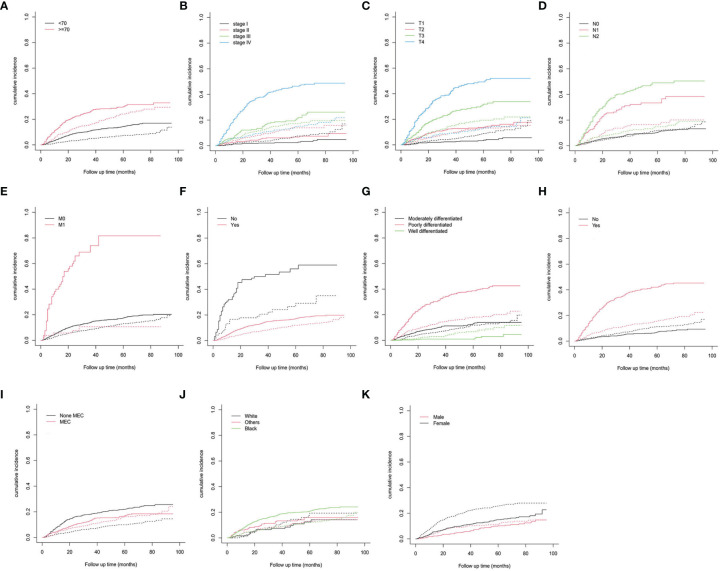
Cumulative incidence predictions of CSD in parotid carcinoma. **(A)** Age; **(B)** AJCC stage; **(C)** T stage; **(D)** N stage; **(E)** M stage; **(F)** surgery; **(G)** grade; **(H)** radiation; **(I)** histologic type; **(J)** race; **(K)** sex. Solid line designates CSD; dotted line designates other causes of death. AJCC, American Joint Committee on Cancer; MEC, mucoepidermoid carcinoma.

The median follow-up time was 43 months (1–95) based on the results of the nomogram **(** **Table 2**). In total, 448 patients (30.5%) had died by the end of follow-up, among which 273 (60.9%) patients died from cancer and 175 (39.1%) patients died from other causes. The 3- and 5-year CSD CIF was 21.4% and 24.1%, respectively. The CIF subgroup assessment data exhibited that high CSD majorly occurred in individuals with PC aged ≥70 years (**Figure 2A**) with advanced AJCC stage (**Figure 2B**), advanced T stage (**Figure 2C**), advanced N stage (**Figure 2D**), along with M1 stage (**Figure 2E**), as well as the patients who did not undergo surgical treatment (**Figure 2F**), radiation treatment (**Figure 2H**), and with undifferentiated/poor grade (**Figure 2G**) and MEC (**Figure 2I**). Nevertheless, no considerable difference in CSD was reported and race and sex subgroup assessments (**Figures 2J, K**).

**Table 2:** Cumulative incidence of cancer-specific death in parotid cancer.

Characteristics	Total number of patients (*n* = 1,467)	Cumulative incidence		*p*-value
		3 years	5 years	
Age (years)				<0.001
<70	980	11.8%	14.3%	
≥70	487	27.9%	29.9%	
Race				0.556
White	130	8.9%	11.1%	
Black	195	17.1%	19.3%	
Others	1,142	13.9%	15.0%	
Sex				0.282
Male	639	10.3%	11.4%	
Female	828	12.4%	14.9%	
AJCC stage				<0.001
I	454	2.0%	2.8%	
II	334	6.1%	6.6%	
III	259	18.0%	21.9%	
IV	420	41.8%	46.4%	
T stage				<0.001
T1	514	2.6%	3.7%	
T2	425	12.7%	13.5%	
T3	292	26.8%	31.8%	
T4	236	44.7%	49.2%	
N stage				<0.001
N0	1,058	8.8%	10.5%	
N1	159	32.1%	33.3%	
N2	250	43.0%	49.0%	
M stage				<0.001
M0	1,414	15.2%	17.5%	
M1	53	74.0%	81.6%	
Surgery				<0.001
No	92	51.6%	55.9%	
Yes	1,375	14.9%	17.1%	
Grade				<0.001
Well	599	0.9%	0.9%	
Moderate	533	11.2%	13.2%	
Poorly/undifferentiated	335	34.3%	38.7%	
Radiation				<0.001
No	946	5.5%	7.1%	
Yes	521	38.2%	42.0%	
Histology type				<0.001
MEC	521	15.1%	17.0%	
No-MEC	846	19.1%	21.9%	

### Nomogram Development

**Figure 3:**
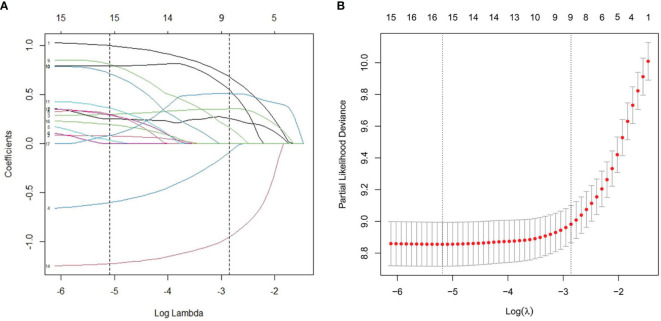
L1-penalized (Lasso) regression model were adopted to determine further predictive variables in test dataset. **(A)** LASSO coefficient patterns of the features. **(B)** Ten-time cross-verification for tuning parameter selection in the Lasso model.

**Figure 4:**
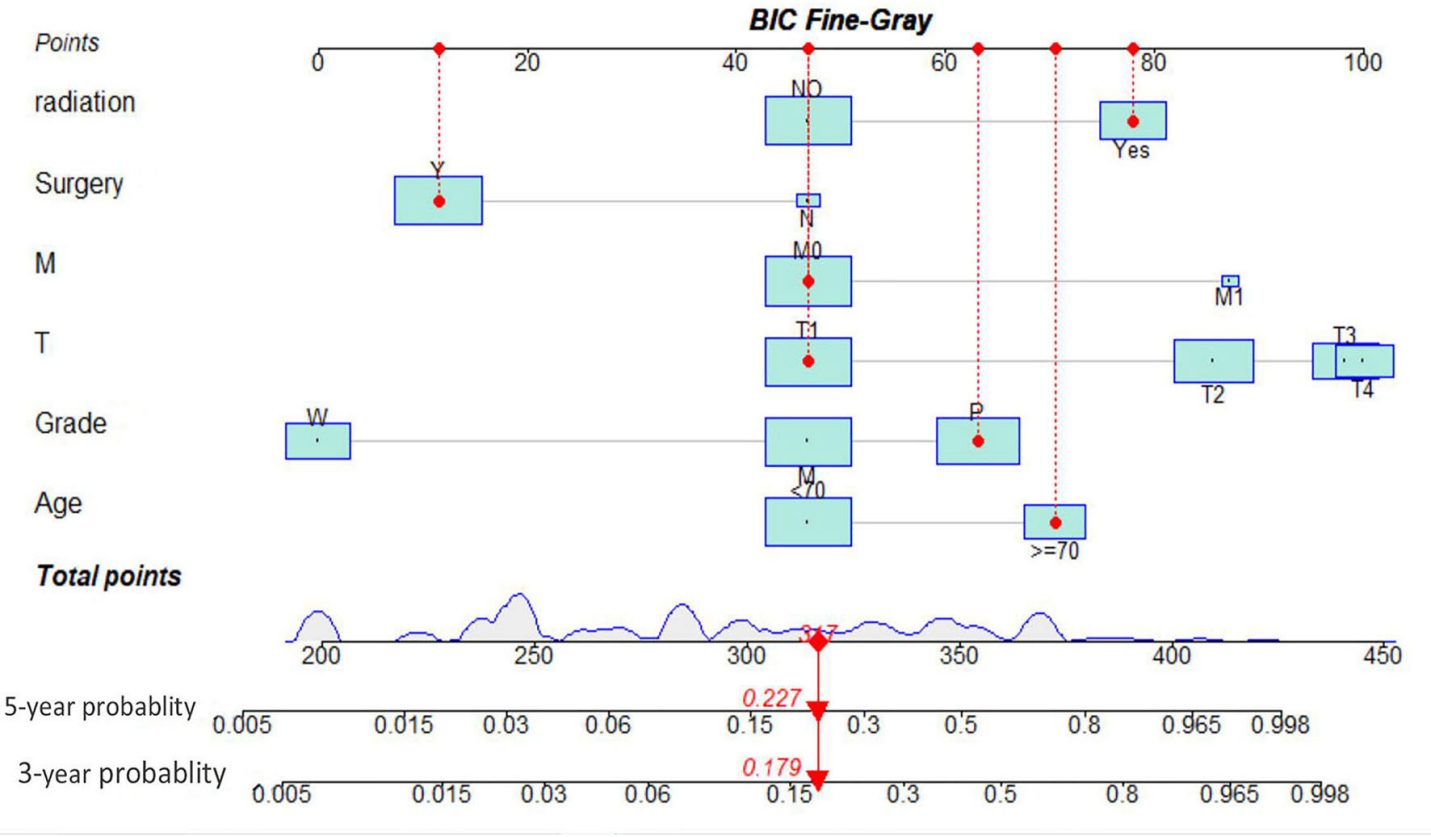
Interactive nomogram for predicting 3- and 5-year likelihoods of CSD in parotid carcinoma. BIC, Bayesian information criterion; MEC, mucoepidermoid carcinoma.

As illustrated in **Table 1**, the patients from the SEER data repository were stratified at random into a test group (*n* = 1,035) and a verification group (*n* = 432) at a ratio of 7:3. We implemented univariate and Lasso Cox assessments in the test dataset to determine independent predictors affecting CDS. A total of nine predictive factors (AJCC stage, surgery, age, T stage, M stage, grade, N stage, histologic, and radiation) were incorporated in the predictive model (**Figures 3A, B**). All variables were further identified by the multivariate assessment of Fine-Gray proportional subdistribution hazards model. As per the AIC assessment, age, T stage, surgery, N stage, histologic, M stage, grade, as well as radiation were independent predictors influencing cancer-distinct death in individuals with PC of the test cohort (*p* < 0.05). Following the optimization of the nomogram as per the BIC, we finally incorporated six variables in the estimation model (**Table 3**). A competing event nomogram was created to assess the 3- and 5-year chances of CSD by using these variables (**Figure 4**). Each patient’s likelihood of death caused by PC at various time points was computed *via* this model through the addition of the scores of each of the integrated variables.

**Table 3:** Results of univariate and multivariate analyses by Fine-Gray proportional subdistribution hazards model in the training cohort.

Characteristics	Univariate analysis		Multivariate analysis (AIC)		Multivariate analysis (BIC)	
	HR (95% CI)	*p*-value	HR (95% CI)	*p*-value	HR (95% CI)	*p*-value
Age (years)						
<70	Ref		Ref		Ref	
≥70	3.274 (2.461–11.718)	<0.001	2.543 (2.082–3.107)	0.001	1.997 (1.53–2.605)	0.003
Race						
Black	Ref					
White	0.991 (0.606–1.833)	0.376228				
Others	0.756 (0.408–1.503)	0.973185				
Sex						
Male	Ref					
Female	1.168 (0.87–2.387)	0.301423				
AJCC stage						
I	Ref					
II	0.595 (0.239–0.969)	0.009				
III	0.57 (0.232–0.974)	<0.001				
IV	0.575 (0.163–0.985)	<0.001				
T stage						
T1	Ref		Ref		Ref	
T2	2.861 (1.326–3.767)	0.008	2.098 (1.176–3.742)	0.012	2.072 (1.262–3.113)	0.090
T3	4.719 (2.298–9.959)	<0.001	3.134 (1.826–5.377)	<0.001	3.374 (1.392–5.919)	<0.001
T4	4.368 (1.945–6.993)	<0.001	3.223 (1.807–5.746)	<0.001	4.716 (2.994–7.885)	<0.001
N stage						
N0	Ref		Ref			
N1	1.352 (1.131–1.88)	<0.001	2.139 (1.3–3.522)	<0.001		
N2	1.865 (1.238–2.554)	0.007	2.36 (1.495–3.726)	<0.001		
M stage						
M0	Ref		Ref		Ref	
M1	2.696 (1.231–3.425)	0.01	2.51 (1.733–3.636)	0.001	2.678 (1.707–4.202)	<0.001
Surgery						
No	Ref		Ref		Ref	
Yes	0.17 (0.09–0.995)	<0.001	0.312 (0.235–0.414)	<0.001	0.338 (0.236–0.482)	<0.001
Radiation						
No	Ref		Ref		Ref	
Yes	2.116 (1.219–2.268)	<0.001	2.139 (1.3–3.522)	<0.001	1.817 (1.117–3.603)	<0.001
Grade						
Well	Ref		Ref		Ref	
Moderately	1.267 (1.061–1.938)	<0.001	1.223 (1.101–3.742)	<0.001	1.684 (1.131–2.132)	<0.001
Poorly/undifferentiated	2.78 (1.847–6.338)	<0.001	2.529 (1.811–3.532)	0.001	1.964 (1.054–3.659)	0.03
Histologic type						
MEC	Ref		Ref			
None MEC	2.602 (1.64–5.155)	<0.001	2.477 (1.715–3.576)	<0.001		

### Nomogram Verification

**Figure 5:**
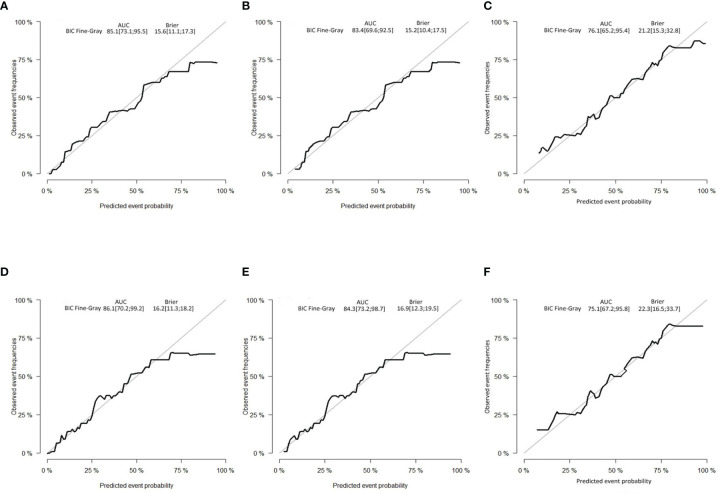
Calibration curves. **(A)** Three-year and **(D)** 5-year likelihoods of CSD in the test dataset. **(B)** Three-year and **(E)** 5-year likelihoods of CSD in the internal verification dataset. **(C)** Three-year and **(F)** 5-year likelihoods of CSD in the external verification dataset. BIC, Bayesian information criterion; AUC, area under the curve.

The C-indexes of the developed nomogram for prediction of the likelihood of CSD in the test data set were 0.862, and the internal verification datasets were 0.843 and 0.795 in the external verification. The AUC of the competing risk nomogram model for forecasting 3- and 5-year likelihoods of CSD was 0.851 and 0.861 in the test cohort, 0.834 and 0.843 in the internal verification cohort, and 0.761 and 0.751 in the external verification cohort. The calibration plots demonstrated optimal consistency of the actual likelihood with the nomogram-forecasted likelihoods in the test (**Figures 5A, D**), as well as verification datasets (**Figures 5B, C, E, F**). The above data illustrated the good estimation potential along with the high confidence of our nomogram.

### Decision Curve Analysis

**Figure 6:**
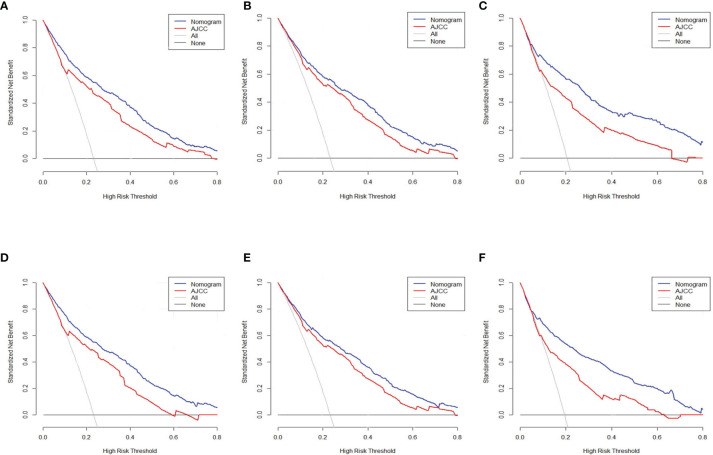
Decision curve assessment of the nomogram along with the AJCC staging approach in the estimation of the CSD of individuals with PC at 3 and 5 years in the test cohort **(A, D)**, internal verification **(B, E)**, and external verification **(C, F)** cohorts.

The DCA was carried out in the test, internal verification, and external verification datasets. The estimation model exhibited an elevated net benefit coupled with a wide range of cutoff likelihood in contrast with the AJCC categorization criteria, illustrating that our prognostic model exhibited a high clinical application value (**Figures 6A–F**).

## Discussion

Salivary gland tumors (SGT) are rare, representing less than 3% of all head and neck tumors (17). On the basis of literature, 22%–35% of SGT are malignant, with the percentage of malignant SGT in the parotid being 15%–25% (1, 18). The pathological types of PC are very complex. Different types of tumors have different clinical and imaging manifestations, treatment, and prognosis. Herein, for the first time, a nomogram for the prognosis of persons with PC was created in a competitive risk nomogram and determined more precise indicators. The large-sized samples available in the SEER data repository reduce errors in this research. Relative to prevailing tools for assessing survival outcomes, the developed nomogram ensures that the chosen variables can be directly associated with a prognosis of cancer. Currently, the most widely used prognostic tool for all solid tumors, including salivary gland tumors, is the TNM staging system (19), but this staging system did not include treatment options such as surgery, chemotherapy, and radiotherapy. However, a nomogram can allow individualized examination of patient prognosis since it incorporates numerous variables.

Of the 11 variables determined in this study, these predictors have been proven in other studies (20, 21). Nine (age, AJCC stage, radiation, T stage, M stage, grade, surgery, N stage, and histological type) were established as indicators of CSD in persons with PC *via* univariate coupled with the Lasso Cox regression model competing risk assessment. Sex and race were excluded in the univariate assessment, illustrating that they do not influence CSD in persons with PC. Assessment of multiple variables using the competing nomogram showed that the AJCC stage was not an independent indicator of patient prognosis. Finally, six variables (age, T stage, M stage, surgery, radiation, and grade) were used to construct the nomogram.

Similar to a study by Sun et al., age was found to independently influence the prognosis of parotid gland mucoepidermoid carcinoma (20). Lyu et al. investigated staging of PC and documented that patient age, favoring 40–60-year-old patients, which is a considerable independent indicator after adjusting for other confounders, might be because older patients have more comorbidities coupled with elevated perioperative risks (22, 23). This finding was congruent with Sun et al. who documented that the prognosis was not remarkably different across races (20) and survival differences between races are not remarkable. Fang et al. documented that sex had no effect on cancer-distinct survival of PC patients, which is congruent with our work (24). The findings also showed that histology type and N stage were independent indicators for PC patients, which is in agreement with previous findings in malignant salivary gland tumor research (25). To prevent overfitting, the aforementioned factors were omitted using PC to improve the performance of the model.

Surgical treatment is the most frequently used therapy for PC at all stages, although according to the guidelines published by the NCCN, surgery is highly recommended for resectable PC (T1–T4a) (26). The data herein demonstrated that surgical therapy could remarkably diminish the tumor-distinct risk of death in individuals with PC; this has been confirmed by most clinicians. However, neck dissection is a controversial subject in parotid malignant tumors. In the presence of a clinically palpable lymph node, there is a consensus on the application of elective neck dissection with a primary parotid surgery (27), some authors support elective neck dissection depending on the tumor histology, size, and grade (28, 29). However, in the study by Ali et al., they suggested that the neck is susceptible to be a target region for metastatic diseases; therefore, complete neck dissection between levels I to V is recommended (30). We established that radiation therapy could remarkably suppress deaths in PC patients, but whether radiotherapy can significantly improve the prognosis of patients is still controversial. In the study by Kaur et al., they believe that postoperative radiotherapy (PORT) has shown a survival benefit in patients with major salivary gland carcinoma (31). For patients with resected T1–2 tumors, the present protocols advocate radiation treatment after operation for individuals with adenoid cystic pathology, close (<1 mm) perineural, or lymphovascular infiltration, or positive margins, lymph node metastasis, as well as intermediate- or high-grade histology (26). In a parotid gland infiltrating ductal carcinoma (IDC) research, they found that PORT only enhanced survival of individuals with parotid gland IDC within T3–4, N1, and TNM III subgroups (32).

Mucoepidermoid carcinoma (MEC), the most prevalent type of PC, constitutes approximately 30–50% of malignant salivary glands (33, 34) However, there is no prognostic analysis of different pathological types of parotid carcinoma. Herein, MEC accounted for 42.3% of all PC cases, and we exhibited that the risk of CSD in persons with MEC was not remarkably higher relative to that in other types of PC such as adenoid cystic carcinoma and polymorphous adenocarcinoma, adenocarcinoma NOS, and myoepithelial carcinoma. This is consistent with the result of Filho OVO et in a retrospective analysis of 193 patients (25). Nevertheless, Baddour et al. and Kokemueller et al. revealed higher survival rates at 5, 10, and 15 years for MEC in relation to other types of PC (35, 36). We think that this should be related to the difference in diagnosis and treatment level between different regions.

Previous investigation on parotid carcinoma based on the SEER data repository focused on incidence, along with survival trends (37, 38), while we focused on creating a prognostic nomogram herein. The clinical therapy of PC and the evaluation of prognosis currently depends on the AJCC staging method. Our prognostic model is suitable for all persons with PC and could be extensively applied in all levels of medical centers. The comprehensive nature of the nomogram may cover the shortcomings of the AJCC staging method, and allow individualized treatment, as well as the precise examination of the prognosis of individuals with PC. Besides, the user-friendly graphic interface of the nomogram could promote the interaction of clinicians with patients. Additionally, a verification data set was utilized for external verification, and the data were drastically congruent with actual survival likelihoods.

However, this research had some shortcomings. In the first place, the SEER data repository lacks some pivotal factors tied to prognosis, including perineural invasion, smoking history, chronic parotitis history, comorbidities, and lack of genetic records of patients. Besides, we adopted the sixth or seventh edition of the AJCC staging method, which lacks two pivotal variables (depth of invasion, as well as an extranodal extension) in contrast with the eighth edition. Moreover, the SEER repository lacks data on tumor volume, which is considered a significant prognostic factor for Salivary gland tumors. Even though this work incorporated the data on chemotherapy and radiotherapy, but the SEER database lacks detailed data on cycles number and doses of chemotherapy, the radiotherapy approaches, and the follow-up treatment after relapse. These variables can also influence the prognosis. Lastly, even though the SEER data repository provided an extensive range of samples for this analysis, errors exist when this database is utilized in the global context. Besides, the data of the external verification are only from a single province in China. It has been reported that even in China, there are different epidemiological differences between the north and the south (1, 39). Therefore larger-sample multicenter study should be conducted to further improve our estimation model and validate its clinical application significance.

## Conclusion

We have created a competing risk nomogram for PC patients using the data retrieved from the SEER data repository and carried out external verification to show the precision and reliability of our nomogram. This well-calibrated nomogram could be utilized in making clinical decisions regarding the prognosis as well as personalized treatment of PC patients.

## Data Availability Statement

The original contributions presented in the study are included in the article/supplementary material. Further inquiries can be directed to the corresponding authors.

## Ethics Statement

Written informed consent was obtained from the individual(s) for the publication of any potentially identifiable images or data included in this article.

## Author Contributions

XL and MH were responsible for the conception and design of the study, assisted with the statistical analysis, and wrote and revised the manuscript. MH and WG were involved in the collecting of data and follow up of the patients. SC and JM contributed their help on the data analysis, revised the English language and grammar, and corrected parts of the discussion. DL has made the primary contribution in the later stage in the writing and modification of the paper and the review of the finalized article. All authors contributed to the article and approved the submitted version.

## Funding

This study was supported by the National Natural Science Foundation of China (No. 81560410) and the Postgraduate Innovation Special Foundation of Jiangxi Province (YC2020-B043).

## Conflict of Interest

The authors declare that the research was conducted in the absence of any commercial or financial relationships that could be construed as a potential conflict of interest.

## Publisher’s Note

All claims expressed in this article are solely those of the authors and do not necessarily represent those of their affiliated organizations, or those of the publisher, the editors and the reviewers. Any product that may be evaluated in this article, or claim that may be made by its manufacturer, is not guaranteed or endorsed by the publisher.
